# Leukocyte count and the risk of adverse outcomes in patients with HFpEF

**DOI:** 10.1186/s12872-021-02142-y

**Published:** 2021-07-07

**Authors:** Zhaowei Zhu, Shenghua Zhou

**Affiliations:** Department of Cardiovascular Medicine, The Second Xiangya Hospital, Central South University, Changsha, 410011 Hunan China

**Keywords:** HFpEF, Leukocyte, Adverse outcomes

## Abstract

**Background:**

Inflammation is a key feature of heart failure including HFpEF. The leukocyte count is a marker of inflammation that is widely used in clinical practice. However, there is little available evidence for the relationship between leukocyte count and the outcomes of HFpEF.

**Methods:**

We analyzed data from the TOPCAT (Treatment of Preserved Cardiac Function Heart Failure with an Aldosterone Antagonist) trial. The primary outcome was all-cause mortality, the secondary outcome was composite cardiovascular events and hospitalization for heart failure. Multivariable Cox proportional hazard models were used to compare the risk profiles of patients with leukocyte quartiles, subgroup study divided by sex was also analyzed.

**Results:**

The present study included 2898 patients with HFpEF.429 deaths, 671 composite cardiovascular events and 386 hospitalization for heart failure occurred during a mean 3.4 years follow-up. The association between leukocyte count and adverse outcomes followed a U-shaped curve. After multivariable adjustment, the patients with the lowest leukocyte count (Q1) and the highest leukocyte count (Q4) faced higher risk of all-cause death(Q1 vs. Q2, adjusted HR: 1.439; 95% CI: 1.060–1.953, p = 0.020; Q4 vs. Q2, adjusted HR, 1.901; 95%CI: 1.424–2.539, p < 0.001). The subgroup analysis showed a consistent result in female but not male patients.

**Conclusions:**

The association between leukocyte count and risk of adverse outcomes followed a U-shaped curve. Both higher and lower leukocyte count are associated with worse outcomes in patients with HFpEF, which may be attributed to the two sides of inflammation in cardiac remodeling.

**Supplementary Information:**

The online version contains supplementary material available at 10.1186/s12872-021-02142-y.

## Background

Heart failure with preserved ejection fraction (HFpEF) has emerged as anpivotal problem with increasing prevalence and poor prognosis in recent years [1]. However, it is still not fully understood of the pathophysiology of HFpEF, which retards the improvement of its accurate diagnosis and efficient treatment.In fact, proven effective medical treatment has not yet appeared for this disease [2, 3].

Leukocyte, as an inflammation driver, plays an important role in cardiovascular disease. In further, it even serves as an important predictor for various cardiovascular events [4–6]. Heart failure, which is an end stage of all kinds of cardiovascular disease, has been known to be involved in inflammation process and the concept of inflammation as a major component of HF is becoming more and more consolidated [7]. Recent studie sconfirmed that inflammatory processes could be part of the etiology of HF [8, 9]. Besides, it was shown that increased long-term incidence of HF hospitalizations were associated with high leukocyte counts [10].Moreover, subclinical inflammation predicts adverse prognosis in patients with established HF [11–13].Canakinumab (IL-1β inhibitor), as an inflammation inhibitor, has beenfound to be capable of reducing not only the incidence of hospitalization for heart failure but also heart failure-related mortality [13].

Although limit evidences indicateinflammation biomarkers are associated with adverse outcomes in patients with HFpEF [14, 15], the relationship between leukocyte count and HFpEF is still not fully clear. Therefore, this study aimed to examine the prognostic significance of leukocyte count on clinicaloutcomes in patients with HFpEF in the Treatment ofPreserved Cardiac Function Heart FailureWith an Aldosterone Antagonist Trial(TOPCAT).

## Methods

### Study design and patients

TOPCAT was a randomized, placebo-control, double blind, multi-centerclinical study.The study aimed to investigate the treatment efficacy of spironolactone in patientswith HFpEF. The study information including background, design, inclusion and exclusion criteria, and baseline characteristicshave been published previously [16, 17]. Briefly, this trial, beginning in August 2006 and ending in January 2012, enrolled 3445 patientswith symptomatic HFpEF from 270 sites distributed in 6 countries. The primary goal of the trial was toclarifywhether spironolactone could reduce the compositeoutcome of aborted cardiac arrest, cardiovascular mortality, orheart failure hospitalization in patients with HFpEF (e.g. documented ejectionfraction ≥ 45%).

**Fig. 1:**
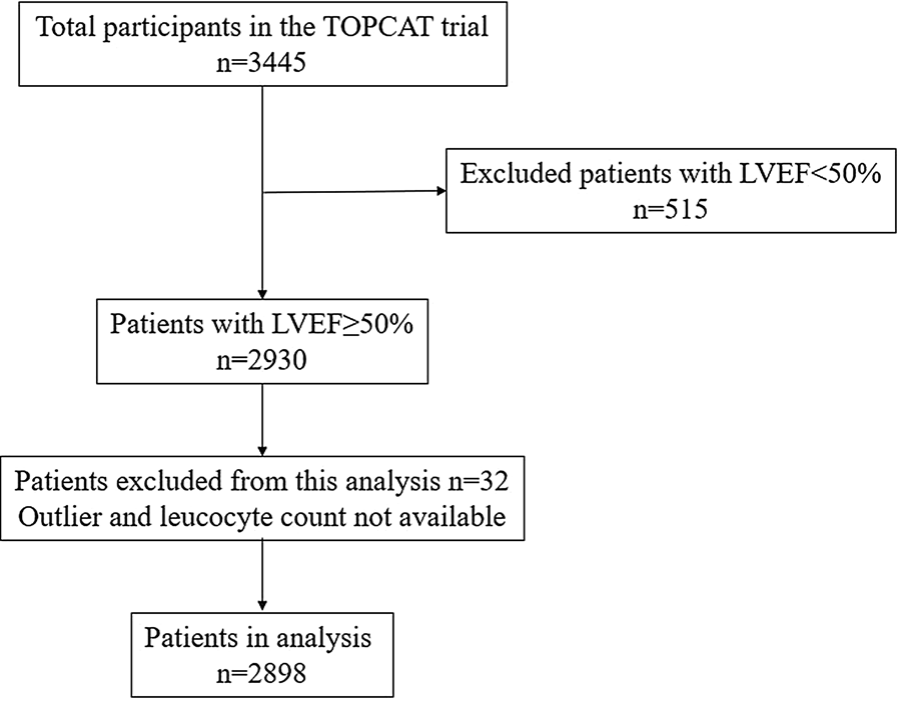
Flow diagram for subject selection

According to the current guideline [18], this analysis in this investigation were limited to patients with ejectionfraction ≥ 50% (n = 2930).Patients with missed leucocyte count and outlier leucocyte count (over 20,000 cells/μL) (n = 32) were excluded. At last, total 2898 patients were enrolled in this study (Fig. 1).The association between leukocyte count on admission and the risk ofall-cause death, the composite cardiovascular events and hospitalization for heart failure were analyzed.

### Baseline characteristics

Basic informationandmedical histories were obtained in patients by a detailed baseline visit in TOPCAT study [17]. For example, age, sex, race, and current smokers were obtained by self-reported history.Medical history included: hypertension, diabetes, stroke, dyslipidemia, peripheral arterial disease, angina pectoris, myocardial infarction, percutaneous coronary revascularization, coronary artery bypass graft surgery, implanted cardioverter defibrillator, implanted pacemaker, thyroid disease, chronic obstructive pulmonary disease, New York HeartAssociation Class, and prior heart failure hospitalization. Systolic bloodpressure, diastolic blood pressure and Body Mass Index (BMI) were obtained by trained staff.Laboratorydata included serum creatinine, blood urea nitrogen (BUN), hematocrit, Brain Natriuretic Peptide (BNP), hemoglobin and platelet. Medication data included: aspirin, angiotensin-converting enzyme inhibitors/angiotensin II receptorblockers, beta blockers, calcium channel blockers, and statins.The National Heart, Lung, and Blood Institute approved our use of TOPCAT data.Ethics approval and consent toparticipate were not applicable.

### Statistics

Baseline characteristics were compared by quartiles of leukocyte counts. Data are presented asmean ± SD,nonnormal variables were reported as median (interquartile range [IQR]—the distance between the 25th and 75th percentiles. Normally distributed continuousvariables were analyzed with one-way ANOVA. Categorical variables were compared withPearson χ^2^ test.Baseline plasma BNP levels were expressed as log-transformed data.Glomerular filtrationrates were estimated by incorporating creatinine into the ChronicKidney Disease Epidemiology Collaboration (CKD-EPI) formula [19].UnadjustedKaplan-Meier estimates of the time-to-event outcomes were generatedaccording to baseline leukocyte countquartiles and compared via the log-rank test.Univariate and multivariable Cox regression analysis were used to test the risk of adverse outcomes associated withleukocyte count. Only variables with p < 0.1 on univariate analysis were incorporated into the multivariate Cox regression analysis. Subgroup analyses of multivariate models were done by sex. Two-sided P-values < 0.05 were consideredstatistically significant. All analyses were performed usingEmpower(R) (www.empowerstats.com, X&Y solutions, IncBoston, MA) andSPSS version 25.0 (IBM, Armonk, New York).

## Results

### Study participants and baseline characteristics

A total of 2898 patients (mean age = 69 ± 9.6 years; 46% men; 89%white) were included in this analysis. Table 1 presented participants’ baseline characteristics based onleukocyte quartiles (Q):Q1: ≦ 5.5 × 10^9^/l; Q2: > 5.5 × 10^9^/l to ≦ 6.7 × 10^9^/l; Q3: > 6.7 × 10^9^/l to ≦ 8.0 × 10^9^/l; and Q4: > 8.0 × 10^9^/l. Leukocyte quartiles were not associated with any significanttrends in age, race, prior heart failure hospitalization, hypertension, stroke, history of pacemaker or implantable cardioverter defibrillators (ICD) implanted,angina pectoris, systolic blood pressure, left ventricular ejection fraction (LVEF), heart rate, the use ofb-blockers, calcium channel blockers, angiotensin-converting enzyme inhibitor/Angiotensin Receptor Blocker (ACEI/ARB)and spironolactone.However, male sex, smoker, dyslipidemia, previous myocardial infarction, percutaneous coronary intervention (PCI), Coronary artery bypass graft (CABG), diabetes mellitus, atrial fibrillation, chronic obstructive pulmonary disease (COPD), asthma, thyroid disease, peripheral arterial disease, use of statins and loop diuretics were more prevalent in participants the higherleukocyte quartiles.At the same time, higher leukocytecount was associated with higher heart rate, body mass index, BUN, hemoglobin and platelet.The higher leukocyte count was also associated with lower diastolic blood pressure, eGFR and prevalence of New York Heart Association class III-IV.

**Table 1:** Baseline characteristics (n = 3421)

Characteristic	Leukocyte count				
	≦ 5.5 n = 753	5.5–6.7 n = 707	6.7–8.0 n = 720	> 8.0 n = 718	p-value
Age, mean ± SD, years	69 ± 9.2	69 ± 9.7	69 ± 10	69 ± 9	0.867
Male (%)	289 (38)	304 (43)	362 (50)	372 (52)	0.000
Race					0.620
White (%)	671 (89)	629 (89)	641 (89)	629 (88)	
Black (%)	69 (9)	58 (8)	63 (9)	66 (9)	
Other (%)	13 (2)	20 (2)	16 (2)	23 (3)	
Smoker (%)	237 (32)	241 (34)	267 (37)	306 (43)	0.001
Hypertension (%)	685 (91)	645 (91)	673 (94)	673 (94)	0.077
Dyslipidemia (%)	431 (57)	406 (57)	423 (59)	483 (67)	0.000
Previous myocardial infarction (%)	143 (19)	154 (22)	173 (24)	192 (27)	0.004
Prior heart failure hospitalization (%)	562 (75)	511 (72)	520 (72)	504 (70)	0.304
Angina pectoris (%)	340 (45)	347 (49)	345 (48)	311 (43)	0.112
PCI (%)	89 (12)	87 (12)	97 (14)	132 (19)	0.000
CABG (%)	75 (10)	80 (11)	85 (12)	113 (16)	0.006
Diabetes mellitus (%)	198 (26)	198 (28)	244 (34)	318 (44)	0.000
Atrial fibrillation (%)	262 (35)	218 (31)	239 (33)	280 (39)	0.011
COPD (%)	58 (8)	67 (10)	89 (12)	124 (17)	0.000
Asthma (%)	36 (5)	56 (8)	43 (6)	61 (9)	0.016
Stroke (%)	56 (7)	43 (6)	59 (8)	68 (10)	0.112
Peripheral arterial disease (%)	49 (7)	55 (8)	66 (9)	89 (12)	0.000
Thyroid disease (%)	128 (17)	105 (15)	104 (15)	143 (20)	0.021
Pacemaker implanted (%)	64 (9)	50 (7)	56 (8)	61 (9)	0.713
ICD (%)	10 (1.3)	8 (1.1)	8 (1.1)	12 (1.7)	0.773
HR (b.p.m.)	69 ± 10.1	68 ± 9.9	68 ± 11.1	70 ± 11.3	0.078
Systolic blood pressure, mean ± SD, mmHg	129 ± 12.6	130 ± 13.9	130 ± 14.6	129 ± 14.9	0.110
Diastolic blood pressure	76 ± 10.4	77 ± 10.6	76 ± 10.8	74 ± 11.1	0.000
Body mass index, mean ± SD, kg/m^2^	31 ± 6.6	32 ± 6.5	32 ± 7.1	34 ± 7.9	0.000
eGFR (mL/min)	67 ± 18.2	69 ± 22.5	68 ± 19.8	65 ± 20.1	0.002
BUN (mg/dL)	16.5 (6.8,22.1)	16.2 (5.0,22.4)	16.5 (5.6,23.0)	17.6 (8.1,26.0)	0.004
Hematocrit (%)	39 ± 5.0	40 ± 4.8	40 ± 5.4	41 ± 5.7	0.000
Hemoglobin (g/dL)	12.9 (12.0,14.0)	13.2 (12.2,14.3)	13.4 (12.3,14.5)	13.5 (12.2,14.8)	0.000
Platelet (k/uL)	207 (173,243)	220 (188,254)	223 (193,264)	245 (208,294)	0.000
Albumin (g/dL)	3.9 ± 2.5	3.8 ± 2.7	3.7 ± 2.5	3.7 ± 2.8	0.000
logBNP	2.6 ± 0.5	2.6 ± 0.5	2.6 ± 0.5	2.6 ± 0.5	0.627
LVEF (%)	59 ± 6.5	59 ± 6.9	59 ± 6.0	59 ± 6.7	0.076
New York Heart Association class III-IV (%)	514 (68)	509 (72)	501 (70)	428 (60)	0.000
Aspirin use (%)	453 (60)	458 (65)	475 (66)	458 (64)	0.110
b-blockers (%)	573 (76)	555 (79)	565 (79)	551 (78)	0.599
ACEi (%)	504 (66)	455 (64)	455 (63)	438 (61)	0.120
ARB (%)	107 (14)	113 (16)	109 (15)	132 (18)	0.155
Statins (%)	334 (44)	332 (47)	362 (50)	426 (59)	0.000
Calcium channel blockers (%)	276 (37)	292 (41)	272 (38)	281 (39)	0.300
Spironolactone (%)	361 (48)	370 (52)	346 (48)	378 (53)	0.118
Loop diuretic (%)	326 (43)	329 (47)	349 (49)	458 (64)	0.000
Thiazide diuretic (%)	322 (43)	278 (39)	286 (40)	216 (30)	0.000

Values are presented as mean ± SD or median (25th-75th percentile) for continuous variables and number (%) for categorical variables. Statistical significance for continuous data was tested using the analysis of variance procedure and categorical data was tested using the χ^2^test

ACEI, angiotensin-converting enzyme inhibitor; ARB, angiotensin receptor blocker; BMI, body mass index; BNP, B-type natriuretic peptide; BUN, blood urea nitrogen; ICD, Implantable Cardioverter Defibrillator; COPD, chronic obstructive pulmonary disease; CABG, Coronary Artery Bypass Grafting;PCI, percutaneous coronary intervention;DBP,diastolic blood pressure; eGFR, estimated glomerular filtration rate; HR, heart rate; LVEF, left ventricular ejection fraction; NYHA, New York Heart Association; SBP, systolic blood pressure

eGFR by the Chronic Kidney Disease Epidemiology Collaboration formula

### Leukocyte count on admission and long-term clinicaloutcomes

**Fig. 2:**
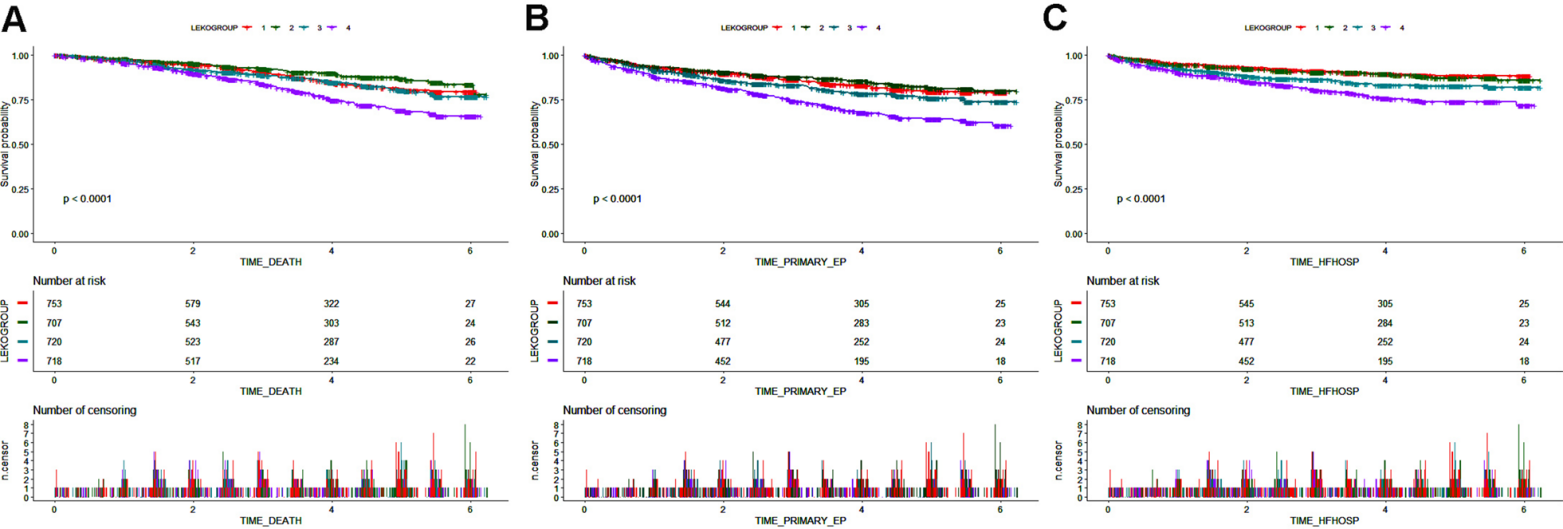
Kaplan–Meier curves of cumulative hazards for adverse cardiovascular outcomes by the leukocyte quartiles

Over a median follow-up of 3.4 years (25th-75thpercentiles = 2.0–4.9 years), 429 deaths, 671 composite cardiovascular events and 386 hospitalization for heart failure occurred. Kaplan–Meier estimates of the cumulative incidence ofall-cause death, the compositecardiovascular eventsand hospitalization for heart failure are depicted in Fig. 2. It seems both participants in the highest and lowest leukocytecount quartiles faced a greater riskfor all-cause death (log-rank, P < 0.0001 forall; Q1 vs. Q2: P < 0.0001; Q3 vs. Q2: P < 0.0001; Q4 vs. Q2: P < 0.0001),compositecardiovascular events(log-rank, P < 0.0001 forall; Q1 vs. Q2: P < 0.0001; Q3 vs. Q2: P < 0.0001; Q4 vs. Q2: P < 0.0001)and hospitalization for heart failure (log-rank, P < 0.0001 forall; Q1 vs. Q2: P < 0.0001; Q3 vs. Q2: P < 0.0001; Q4 vs. Q2: P = 0.003).

**Fig. 3:**
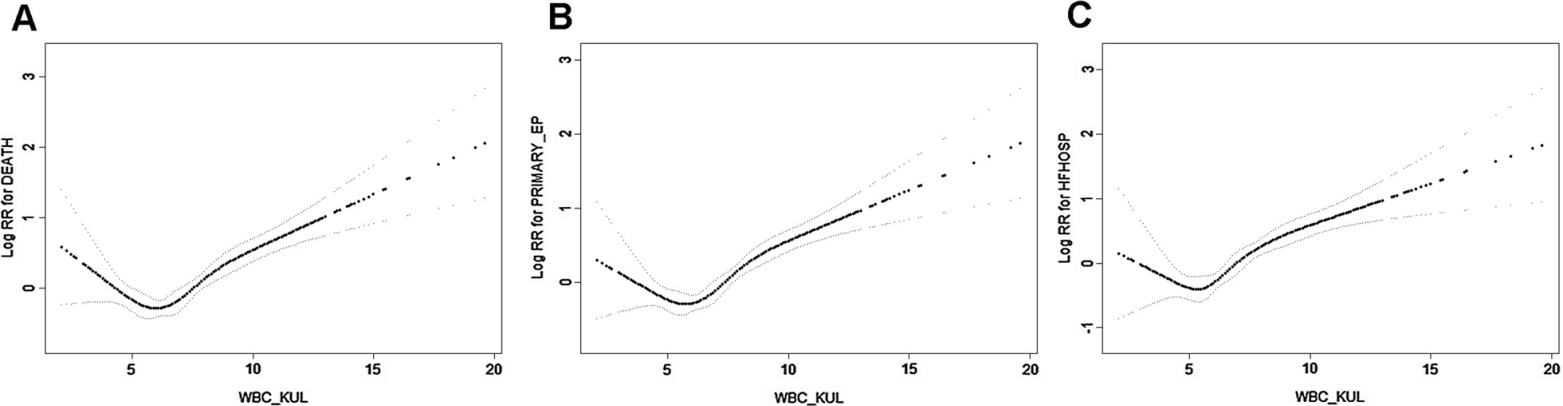
Restricted Cubic Spline of the Association of leukocyte With Risk of adverse cardiovascular outcomes in HFpEF. This figure is as the Central Illustration of our study, which shows a U-shaped relationship between leukocyte count and adverse outcomes in patients with HFpEF.

Actually, the associationbetween leukocyte count and risk of adverse outcomes followed a U-shaped curve, with increased risk above and below the reference range of 5.5 to 6.7 × 10^9^/l(Q2) (Fig. 3).The results of the Cox proportional hazards models illustrating the relationshipbetween leukocyte countand long-term clinical outcomes are shown in Table 2 and Additional file 1: Table S1–S4. As shown in Table 2, leukocyte count was an independent risk factor for all-cause death after multivariable adjustment (P < 0.001). And the participants with the lowest leukocyte count (Q1) and the highest leukocyte count(Q4) had higher risk of all-cause death compared with participants with leukocyte count range from 5.5 × 10^9^/l to 6.7 × 10^9^/l.(Q1 vs. Q2: adjusted HR1.439, 95%CI:1.060 to 1.953, P = 0.020; Q4 vs. Q2: adjusted HR1.901, 95%CI:1.424 to 2.539, P < 0.001).

**Table 2:** Univariate and multivariable Cox regression analysis of all-cause mortality (n = 2898)

All-cause mortality	Univariate analysis			Multivariate analysis		
	HR	95%CI	p-value	HR	95%CI	p-value
Age	1.054	1.043–1.065	0.000	1.046	1.033–1.059	0.000
Sex	0.67	0.554–0.810	0.000	1.698	1.368–2.106	0.000
Race	1.528	1.251–1.867	0.000			0.037
				0.531	0.326–0.865	0.011
				0.591	0.332–1.049	0.073
BMI	1.007	0.993–1.021	0.330	–	–	–
Smoker	1.170	1.037–1.320	0.011	–	–	–
LVEF	0.998	0.984–1.013	0.820	–	–	–
Angina pectoris	0.613	0.504–0.745	0.000	0.815	0.653–1.017	0.071
Prior heart failure hospitalization	0.810	0.657–0.997	0.047	1.124	0.901–1.403	0.301
Previous myocardial infarction	1.266	1.026–1.563	0.028	0.777	0.603–1.002	0.052
Stroke	1.558	1.151–2.110	0.004	0.940	0.686–1.289	0.702
CABG	1.655	1.293–2.118	0.000	1.066	0.800–1.422	0.661
PCI	1.483	1.161–1.893	0.002	1.061	0.803–1.403	0.677
COPD	1.629	1.257–2.111	0.000	0.936	0.713–1.228	0.634
Asthma	1.601	1.152–2.226	0.005	0.812	0.574–1.148	0.239
Hypertension	0.815	0.586–1.133	0.223	–	–	–
Peripheral arterial disease	2.154	1.669–2.779	0.000	0.615	0.468–0.809	0.001
Dyslipidemia	1.271	1.043–1.550	0.018	1.105	0.857–1.426	0.441
ICD	1.605	0.797–3.230	0.185	–	–	–
Pacemaker	1.983	1.500–2.621	0.000	0.978	0.724–1.320	0.884
Atrial fibrillation	1.530	1.264–1.851	0.000	1.016	0.821–1.258	0.884
Thyroid disease	1.219	0.957–1.553	0.108	–	–	–
Diabetes mellitus	0.595	0.491–0.721	0.000	0.857	0.685–1.071	0.175
Heart rate	1.017	1.008–1.026	0.000	1.021	1.012–1.031	0.000
Systolic blood pressure	0.981	0.974–0.988	0.000	0.992	0.984–1.000	0.050
Diastolic blood pressure	0.959	0.951–0.967	0.000	0.994	0.982–1.006	0.300
Fasting glucose	1.002	0.998–1.005	0.343	–	–	–
New York Heart Association class III-IV	1.723	1.423–2.086	0.000	0.806	0.658–0.988	0.038
eGFR	0.979	0.973–0.984	0.000	0.994	0.988–1.000	0.055
Leukocyte group	1.249	1.146–1.361	0.000			0.000
1				1.439	1.060–1.953	0.020
2				Reference		
3				1.510	1.113–2.050	0.008
4				1.901	1.424–2.539	0.000
Hemoglobin	0.833	0.786–0.882	0.000	0.898	0.843–0.958	0.001
BUN	1.030	1.025–1.036	0.000	1.009	1.001–1.017	0.023
Albumin	0.983	0.945–1.023	0.411	–	–	–
Aspirin	1.301	1.074–1.576	0.007	1.089	0.884–1.341	0.424
b-blockers	1.16	0.915–1.471	0.220	–	–	–
ACEi	1.355	1.116–1.643	0.002	0.945	0.770–1.160	0.591
ARB	0.862	0.670–1.109	0.248	–	–	–
Statin	0.726	0.599–0.878	0.001	1.072	0.837–1.372	0.581
Loop diuretic	0.304	0.245–0.377	0.000	0.553	0.423–0.724	0.000
Thiazide Diuretic	0.494	0.398–0.612	0.000	1.080	0.840–1.388	0.548
Spironolactone	1.029	0.851–1.243	0.769	–	–	–

CI: confidence interval; HR: hazard ratio

Interestingly, subgroup analyses of female participants confirmed the U-shaped relationship between leukocyte count and all-cause death (Table 3, P = 0.002). However, despite a similar trend in male participants, there is no significant difference between groups. The subgroup analysis indicated the prognostic value of leukocyte count for all-cause death maybe different in different sexs. And female may contribute more to the relationship between leukocyte count and all-cause death.

**Table 3:** Subgroup analysis of Cox proportional-hazards model divided by sex for All-cause mortality

All-cause mortality	Male			Female		
	HR	95%CI	p-value	HR	95% CI	p-value
Age	1.047	1.029–1.066	0.000	1.038	1.019–1.057	0.000
Race			0.473			0.007
	0.684	0.328–1.424	0.310	0.344	0.175–0.676	0.002
	0.837	0.354–1.982	0.687	0.319	0.143–0.709	0.005
Smoker	0.858	0.743–0.991	0.037	0.864	0.697–1.070	0.180
Angina pectoris	0.967	0.715–1.309	0.830	0.692	0.493–0.970	0.033
Prior heart failure hospitalization	1.269	0.933–1.727	0.130	0.928	0.665–1.296	0.661
Previous myocardial infarction	0.742	0.536–1.025	0.071	0.893	0.583–1.366	0.602
Stroke	0.851	0.551–1.315	0.468	0.993	0.619–1.593	0.978
CABG	1.109	0.772–1.592	0.576	0.953	0.580–1.566	0.850
PCI	1.168	0.805–1.693	0.414	0.927	0.594–1.447	0.739
COPD	1.118	0.780–1.602	0.544	0.687	0.445–1.061	0.091
Asthma	0.602	0.356–1.018	0.058	1.070	0.660–1.736	0.783
Peripheral arterial disease	0.562	0.394–0.801	0.001	0.657	0.420–1.029	0.067
Dyslipidemia	1.158	0.816–1.643	0.412	1.121	0.770–1.634	0.550
Pacemaker	0.863	0.574–1.298	0.479	1.116	0.697–1.787	0.647
Atrial fibrillation	1.139	0.852–1.521	0.380	0.860	0.622–1.189	0.360
Diabetes mellitus	0.986	0.729–1.334	0.926	0.737	0.527–1.032	0.075
Heart rate	1.019	1.006–1.032	0.005	1.028	1.014–1.042	0.000
Systolic blood pressure	0.993	0.982–1.005	0.244	0.994	0.982–1.005	0.286
Diastolic blood pressure	0.990	0.974–1.007	0.251	0.992	0.975–1.010	0.332
New York Heart Association class III-IV	0.805	0.604–1.072	0.138	0.756	0.557–1.026	0.072
eGFR	0.995	0.986–1.003	0.211	0.993	0.984–1.002	0.143
Leukocyte group			0.088			0.002
1	1.134	0.745–1.726	0.557	1.907	1.188–3.059	0.007
2	reference					
3	1.150	0.768–1.721	0.498	2.088	1.291–3.375	0.003
4	1.571	1.071–2.303	0.021	2.445	1.543–3.875	0.000
Hemoglobin	0.889	0.816–0.968	0.007	0.910	0.822–1.006	0.066
BUN	1.011	1.001–1.021	0.032	1.005	0.993–1.018	0.419
Aspirin	1.354	1.021–1.795	0.035	0.838	0.609–1.153	0.277
ACEi	1.007	0.759–1.335	0.963	0.884	0.650–1.202	0.432
Statin	1.006	0.713–1.420	0.972	1.142	0.790–1.651	0.479
Loop Diuretic	0.627	0.441–0.892	0.010	0.467	0.308–0.707	0.000
Thiazide Diuretic	0.925	0.666–1.285	0.642	1.303	0.880–1.930	0.186

After multivariable adjustment (Additional file 1: Table 1), therisk of compositecardiovascular events increased in patients withleukocyte count at Q3(HR, 1606; 95%CI, 1.407to 1.904), Q4(HR, 1.650; 95%CI, 1.108to2.459) compared with patients with leukocyte count at Q2. Although similar trend was found in patients with leukocyte count at Q1, there was no statistical difference. Subgroup analysis by sex only found similar trend without statistical significance (Additional file 1: Table 2).Besides, after multivariable adjustment, participants with higher or lower leukocyte count at Q4 or Q1 did not have an increased risk for hospitalization for heart failure compared with patients with leukocyte count at Q2, and subgroup analysis reach a consistent result (Additional file 1: table s3 and table s4). Above results indicated that leukocyte count was not a prognostic factor for compositecardiovascular events and hospitalization for heart failure.

## Discussion

This study found that the associationbetween leukocyte count and the risk ofadverse outcomes followed a U-shaped curve. Both lower and higher leukocyte count is related to a higher risk of adverse outcomes in the TOPCAT patientscohort.

Several studies have reported that pro-inflammatory biomarkers including high sensitivity C-reactive protein, tumor necrosis factor-α, interleukin 6/8, monocyte chemoattractant protein-1 and pentraxin 3 were significantly increased in patients with HFpEF [14, 20–22].Consistent with previous studies, our results once again confirm that inflammatory responses may play an important role in the progression and development of HFpEF [20, 21, 23].

However, although leukocyte count acts as an important marker for inflammation level in body, few previous studies have assessed the association between leukocyte countand cardiovascular events in patients with HFpEF.Previousstudies only showed that the prognosticvalue of relative lymphocyte count in patients with chronic HFrEF [12, 24–26].In further, high leukocyte countwas found to be associated with increased long-term incidence of HFhospitalizationsin middle-aged men [10].Besides, Kim et al. found that neutrophil-to-lymphocyte ratiowas prospectively associatedwith heart failure [5]. In line with above studies, present finding indicates that leukocyte countisassociated with both all-causedeath and composite cardiovascular events specifically in HFpEF patients, reaffirming this important link between leukocytecount and heart failure regardless of ejection fraction. Recently, Bajaj NS et al. [27]did a similar study and they found that leucocyte count > 7100 cells/μL was independently associated with adverse clinical outcomes especially HF hospitalization in HFpEF patients from TOPCAT-Americas.In our study, we focused on the whole population in TOPCAT study and patients with LVEF < 50% were excluded, which may be attributed to the different result from the study by Bajaj NS. In our study, we found a U-shaped relationship between the risk of clinical outcomes especially all-cause death and leukocyte count. Besides, the subgroup analysis showed that female may contribute more to such relationship of leukocyte count and all-cause death. However, the U-shaped relationship also showed an increased risk of clinical outcomes for patients with higher leukocyte count in our study, which was confirmed by the study by Bajaj NS.Besides, although similar trend was found, leukocyte count was not a prognostic factor for compositecardiovascular events and hospitalization for heart failure in this study. This may be caused by the heterogeneity of HFpEF, the shortage of the second analysis and the limit sample volume. Further well-designed study was warranted to investigate the actual role of leukocyte in patients with HFpEF.

Although the association between leukocyte and heart failure is strongly supported by current clinical evidences [26]. It is not known whether leukocytes are involved directly in the pathogenesis of heart failure or areonly accompany with the disease.Severalsystemic proinflammatory conditions including obesity, hypertension, diabetes or metabolic syndrome were usually combined in patients with HFpEF,whichmight be the fundamental mechanism that leads to inflammation andoxidative stress [28]. The increased pro-inflammatory state and oxidativestress may in turn result incoronary microvascular endothelial dysfunction and myocardialfibrosis, consequently leading to adverse cardiovascular events finally. This may explain the increased risk of adverse outcomes ofHFpEF patients with higher level of leukocyte count in this study.

However, in our study, we presented a U-shaped relationship between leukocyte count and the risk of adverse outcomes, indicatingmore complex mechanisms might be involved underling the relationship between leukocyte level and cardiovascular outcomes in HFpEF patients. Leukocytescan not only facilitate the proteolysis of the collagen matrix but also promote interstitial myocardial fibrosis, which eventually contribute tothe cardiac remodeling and heart failure [4]. Confirming this,recent study demonstratedthat by activating fibroblasts and stimulating collagen deposition, IL-10 derived from T cellsand macrophagescan induce myocardial stiffness and impair myocardial relaxation [29, 30]. But on the other hand, through secretion of angiogenesis-promoting cytokines, leukocytescan also protect the nonischemic remote myocardium in ischemic heart disease [4]. This indicates thattoo lessleukocyte may be harmful for some heart disease.

In addition, the U-shaped relationship between leukocyte count and the risk of adverse cardiovascular outcomes persisted even aftercontrolling for baseline covariates.The U-shaped relationship may also be a potential reason for the unsuccessful clinical trials attempting to combat HFby blocking inflammation [11]. Although canakinumabis related to a dose-dependent reduction in heart failure relatedhospitalization and the composite of heart failure-related mortality and hospitalization, it is not efficient in all population but patients with elevatedhsCRP [31].Besides,interaction between inflammation and body weight, blood pressure, and blood glucose might jointly affect theoutcomes of HFpEF patients and the sum of the complex interaction may bealso responsible for the observedU-shaped relationshipin this study [32–35].

## Conclusions

In this study, we found a U-shaped relationship between leukocyte count and risk of clinical outcomes, and subgroup analysis showed that female contributed more to such relationship for all-cause death. Both higher and lower leukocyte count are associated with worse outcomes in patients with HFpEF, which may be attributed to the two sides of inflammation in cardiac remodeling.

## Limitations

The findings of this study must be interpreted in the contextof limitations inherent to the TOPCAT studydesign. First, there is heterogeneityin HFpEF,so these findings may not represent all theHFpEF classifications. Secondly, we cannot exclude biasintroduced by leukocyte levels measured at laboratories and there is lack of CRP value and serial measurements about leukocyte count in the database, which limit the strength of the conclusion.Thirdly, leukocyte count is elevated or decreased commonly in patient with acute infection or blood system diseases, no information is applied about the exclusion of such patients in the TOPCAT trial, the impact of acute infection or blood system diseases thus remain unknown and served as a limitation of present analysis.At last, although the subtype of leukocyte may play pivotal role in cardiovascular disease, we did not assess the specific role due to the unavailability of the related information in the present database.

## Supplementary Information

**Additional file 1.** **Supplemental Table 1.** Univariate and multivariable Cox regression analysis of Composite cardiovascular events (n = 2898). **Supplemental Table 2.** Subgroup analysis of Cox proportional-hazards model divided by gender for Composite cardiovascular events (n = 2898). **Supplemental Table 3.** Univariate and multivariable Cox regression analysis of hospitalization for heart failure (n = 2898). **Supplemental Table 4.** Subgroup analysis of Cox proportional-hazards model divided by gender for Hospitalization for heart failure (n = 2898).

## Data Availability

The datasets used and/or analysed during the current study are available from the corresponding author on reasonable request.

## Abbreviations

HFpEF: Heart failure with preserved ejection fraction
BMI: Body mass index blood
BUN: Urea nitrogen
BNP: Brain natriuretic peptide
CKD-EPI: Chronickidney disease epidemiology collaboration
ACEI/ARB: Angiotensin-converting enzyme inhibitor/Angiotensin receptor blocker
ICD: Implantable cardioverter defibrillators
PCI: Percutaneous coronary intervention
CABG: Coronary artery bypass graft
COPD: Chronic obstructive pulmonary disease
LVEF: Left ventricular ejection fraction
