# Supplementary material for: Leukocyte count and the risk of adverse outcomes in patients with HFpEF
Source: BMC Cardiovasc Disord. 2021 Jul 7;21:333. doi: 10.1186/s12872-021-02142-y (PMC8261982; doi:10.1186/s12872-021-02142-y)
Supplement: Supplementary file 1 — Additional file 1. Supplemental Table 1. Univariate and multivariable Cox regression analysis of Composite cardiovascular events (n = 2898). Supplemental Table 2. Subgroup analysis of Cox proportional-hazards model divided by gender for Composite cardiovascular events (n = 2898). Supplemental Table 3. Univariate and multivariable Cox regression analysis of hospitalization for heart failure (n = 2898). Supplemental Table 4. Subgroup analysis of Cox proportional-hazards model divided by gender for Hospitalization for heart failure (n = 2898). [file 12872_2021_2142_MOESM1_ESM.docx]

**Supplemental data**

**Supplemental Table 1. Univariate and multivariable Cox regression analysis of Composite cardiovascular events. (n=2898)**

| Composite cardiovascular events | Univariate analysis | | | Multivariate analysis | | |
| --- | --- | --- | --- | --- | --- | --- |
|  | HR | 95%CI | p-value | HR | 95%CI | p-value |
| Age | 1.034 | 1.025-1.043 | 0.000 | 1.025 | 1.007-1.044 | 0.006 |
| Gender | 1.361 | 1.151-1.608 | 0.000 | 1.846 | 1.334-2.553 | 0.000 |
| Race | 1.906 | 1.641-2.214 | 0.000 |  |  | 0.000 |
|  |  |  |  | 0.267 | 0.138-0.516 | 0.000 |
|  |  |  |  | 0.654 | 0.294-1.455 | 0.297 |
| BMI | 1.038 | 1.027-1.049 | 0.000 | 0.991 | 0.970-1.012 | 0.399 |
| Smoker | 1.414 | 1.256-1.591 | 0.000 | 0.961 | 0.574-1.609 | 0.740 |
| LVEF | 1.000 | 0.987-1.013 | 0.985 | - | - | - |
| Angina pectoris | 1.590 | 1.339-1.888 | 0.000 | 1.315 | 0.971-1.781 | 0.077 |
| Prior heart failure hospitalization | 1.071 | 0.888-1.292 | 0.473 | - | - | - |
| Previous myocardial infarction | 0.863 | 0.714-1.044 | 0.130 | - | - | - |
| Stroke | 0.628 | 0.481-0.820 | 0.001 | 0.988 | 0.642-1.520 | 0.955 |
| CABG | 0.526 | 0.426-0.650 | 0.000 | 0.886 | 0.585-1.343 | 0.568 |
| PCI | 0.578 | 1.161-1.893 | 0.002 | 1.030 | 0.673-1.577 | 0.891 |
| COPD | 1.629 | 0.471-0.711 | 0.000 | 0.936 | 0.713-1.228 | 0.634 |
| Asthma | 0.521 | 0.398-0.683 | 0.000 | 0.925 | 0.513-1.670 | 0.797 |
| Hypertension | 0.782 | 0.553-1.106 | 0.164 | - | - | - |
| Peripheral arterial disease | 0.532 | 0.420-0.674 | 0.000 | 0.490 | 0.330-0.728 | 0.000 |
| Dyslipidemia | 0.587 | 0.488-0.705 | 0.000 | 0.791 | 0.568-1.101 | 0.164 |
| ICD | 0.720 | 0.372-1.390 | 0.327 | - | - | - |
| Pacemaker | 2.188 | 1.720-2.785 | 0.000 | 0.962 | 0.570-1.624 | 0.886 |
| Atrial fibrillation | 1.603 | 1.356-1.896 | 0.000 | 0.799 | 0.597-1.070 | 0.133 |
| Thyroid | 1.131 | 0.909-1.407 | 0.270 | - | - | - |
| Diabetes mellitus | 2.359 | 1.996-2.788 | 0.000 | 0.779 | 0.560-1.084 | 0.138 |
| Heart rate | 1.015 | 1.007-1.023 | 0.000 | 1.020 | 1.007-1.033 | 0.002 |
| Systolic blood pressure | 0.990 | 0.984-0.996 | 0.001 | 1.006 | 0.994-1.017 | 0.341 |
| Diastolic blood pressure | 0.954 | 0.947-0.961 | 0.000 | 0.975 | 0.959-0.992 | 0.003 |
| Fasting glucose | 1.005 | 1.003-1.008 | 0.000 | 1.003 | 1.000-1.005 | 0.079 |
| New York Heart Association class III-IV | 2.239 | 1.894-2.647 | 0.000 | 0.592 | 0.444-0.790 | 0.000 |
| eGFR | 0.979 | 0.974-0.984 | 0.000 | 0.996 | 0.988-1.004 | 0.344 |
| Leukocyte group | 1.285 | 1.191-1.387 | 0.000 |  |  | 0.071 |
| 1 |  |  |  | 1.441 | 0.605-1.262 | 0.080 |
| 2 |  |  |  | reference |  |  |
| 3 |  |  |  | 1.606 | 1.407-1.904 | 0.024 |
| 4 |  |  |  | 1.650 | 1.108-2.459 | 0.014 |
| Hemoglobin | 0.782 | 0.744-0.823 | 0.000 | 0.968 | 0.892-1.051 | 0.436 |
| BUN | 1.037 | 1.032-1.042 | 0.000 | 1.013 | 1.002-1.024 | 0.017 |
| Albumin | 0.942 | 0.913-0.972 | 0.000 | 0.983 | 0.937-1.031 | 0.480 |
| Aspirin | 0.863 | 0.727-1.024 | 0.091 | 1.040 | 0.772-1.402 | 0.797 |
| b-blocker | 1.421 | 1.140-1.771 | 0.002 | 0.678 | 0.489-0.940 | 0.020 |
| ACEi | 0.707 | 0.597-0.838 | 0.000 | 1.069 | 0.760-1.504 | 0.700 |
| ARB | 1.618 | 1.321-1.981 | 0.000 | 1.102 | 0.711-1.710 | 0.663 |
| Statin | 1.801 | 1.516-2.139 | 0.000 | 1.229 | 0.881-1.715 | 0.225 |
| Loop Diuretic | 0.230 | 0.188-0.281 | 0.000 | 0.576 | 0.406-0.818 | 0.002 |
| Thiazide Diuretic | 0.483 | 0.399-0.585 | 0.000 | 1.105 | 0.802-1.523 | 0.541 |
| Spironolactone | 1.077 | 0.911-1.273 | 0384 | - | - | - |

**Supplemental Table 2. Subgroup analysis of Cox proportional-hazards model divided by gender for Composite cardiovascular events. (n=2898)**

| Composite cardiovascular events | male | | | female | | |
| --- | --- | --- | --- | --- | --- | --- |
|  | HR | 95%CI | p-value | HR | 95%CI | p-value |
| Age | 1.029 | 1.004-1.054 | 0.025 | 1.011 | 0.982-1.041 | 0.465 |
| Gender | 1.361 | 1.151-1.608 | 0.000 | 1.846 | 1.334-2.553 |  |
| Race |  |  | 0.001 |  |  | 0.074 |
|  | 0.358 | 0.143-0.895 | 0.028 | 0.280 | 0.093-0.838 | 0.023 |
|  | 1.440 | 0.459-4.513 | 0.532 | 0.351 | 0.100-1.231 | 0.102 |
| BMI | 0.987 | 0.958-1.018 | 0.409 | 0.987 | 0.957-1.018 | 0.410 |
| Smoker | 0.962 | 0.525-1.761 | 0.098 | 0.981 | 0.321-3.002 | 0.973 |
| Angina pectoris | 1.147 | 0.761-1.729 | 0.512 | 1.768 | 1.093-2.861 | 0.020 |
| Stroke | 1.348 | 0.714-2.545 | 0.358 | 0.683 | 0.355-1.313 | 0.253 |
| CABG | 0.728 | 0.446-1.190 | 0.205 | 1.467 | 0.599-3.592 | 0.402 |
| PCI | 1.211 | 0.688-2.133 | 0.507 | 1.019 | 0.486-2.134 | 0.961 |
| COPD | 0.598 | 0.368-0.972 | 0.038 | 1.188 | 0.487-2.896 | 0.705 |
| Asthma | 1.012 | 0.443-2.309 | 0.978 | 0.832 | 0.331-2.094 | 0.697 |
| Peripheral arterial disease | 0.374 | 0.230-0.608 | 0.000 | 0.660 | 0.293-1.490 | 0.317 |
| Dyslipidemia | 1.157 | 0.734-1.823 | 0.531 | 0.469 | 0.278-0.792 | 0.005 |
| Pacemaker | 0.585 | 0.319-1.072 | 0.083 | 3.925 | 0.900-17.111 | 0.069 |
| Atrial fibrillation | 0.921 | 0.625-1.358 | 0.578 | 0.584 | 0.369-0.924 | 0.022 |
| Diabetes mellitus | 0.803 | 0.519-1.244 | 0.326 | 0.682 | 0.398-1.168 | 0.163 |
| Heart rate | 1.018 | 1.000-1.036 | 0.047 | 1.035 | 1.014-1.057 | 0.001 |
| Systolic blood pressure | 1.006 | 0.989-1.024 | 0.497 | 1.010 | 0.993-1.028 | 0.250 |
| Diastolic blood pressure | 0.981 | 0.957-1.005 | 0.119 | 0.954 | 0.929-0.979 | 0.000 |
| Fasting glucose | 1.004 | 1.000-1.008 | 0.028 | 1.001 | 0.996-1.005 | 0.815 |
| New York Heart Association class III-IV | 0.708 | 0.468-1.071 | 0.102 | 0.446 | 0.282-0.706 | 0.001 |
| eGFR | 0.995 | 0.984-1.005 | 0.296 | 0.996 | 0.983-1.009 | 0.563 |
| Leukocyte group |  |  | 0.471 |  |  | 0.395 |
| 1 | 1.131 | 0.654-1.957 | 0.660 | 1.573 | 0.534-1.703 | 0.176 |
| 2 | indicator |  |  |  |  |  |
| 3 | 1.274 | 0.742-2.186 | 0.380 | 1.741 | 0.878-3.451 | 0.112 |
| 4 | 1.483 | 0.886-2.481 | 0.134 | 1.655 | 0.844-3.244 | 0.143 |
| Hemoglobin | 0.980 | 0.882-1.089 | 0.710 | 0.958 | 0.828-1.108 | 0.563 |
| BUN | 1.018 | 1.004-1.032 | 0.011 | 1.008 | 0.991-1.025 | 0.371 |
| Albumin | 0.995 | 0.934-1.061 | 0.884 | 0.989 | 0.912-1.074 | 0.799 |
| Aspirin | 1.158 | 0.770-1.743 | 0.480 | 0.885 | 0.552-1.419 | 0.612 |
| b-blocker | 0.647 | 0.415-1.008 | 0.054 | 0.638 | 0.371-1.096 | 0.103 |
| ACEi | 0.945 | 0.590-1.515 | 0.815 | 1.137 | 0.675-1.913 | 0.630 |
| ARB | 0.758 | 0.431-1.336 | 0.338 | 2.055 | 0.952-4.435 | 0.066 |
| Statin | 0.990 | 0.623-1.571 | 0.965 | 1.765 | 1.063-2.930 | 0.028 |
| Loop Diuretic | 0.575 | 0.361-0.918 | 0.020 | 0.577 | 0.329-1.013 | 0.056 |
| Thiazide Diuretic | 0.984 | 0.644-1.502 | 0.940 | 1.179 | 0.694-2.001 | 0.542 |

**Supplemental Table 3. Univariate and multivariable Cox regression analysis of hospitalization for heart failure. (n=2898)**

| Hospitalization for heart failure | Univariate analysis | | | Multivariate analysis | | |
| --- | --- | --- | --- | --- | --- | --- |
|  | HR | 95%CI | p-value | HR | 95%CI | p-value |
| Age | 1.034 | 1.023-1.045 | 0.000 | 1.011 | 0.987-1.036 | 0.360 |
| Gender | 0.790 | 0.647-0.965 | 0.021 | 1.719 | 1.123-2.632 | 0.013 |
| Race | 1.988 | 1.675-2.360 | 0.000 |  |  | 0.015 |
|  |  |  |  | 0.327 | 0.132-0.810 | 0.016 |
|  |  |  |  | 0.636 | 0.224-1.807 | 0.395 |
| BMI | 1.051 | 1.038-1.064 | 0.000 | 1.010 | 0.983-1.037 | 0.486 |
| Smoker | 1.643 | 1.412-1.913 | 0.000 | 1.058 | 0.497-2.254 | 0.840 |
| LVEF | 1.001 | 0.986-1.017 | 0.876 | - | - | - |
| Angina pectoris | 0.551 | 0.446-0.679 | 0.000 | 1.705 | 1.113-2.612 | 0.014 |
| Prior heart failure hospitalization | 0.867 | 0.696-1.079 | 0.201 | - | - | - |
| Previous myocardial infarction | 1.060 | 0.840-1.337 | 0.623 | - | - | - |
| Stroke | 1.446 | 1.042-2.007 | 0.027 | 1.079 | 0.605-1.925 | 0.796 |
| CABG | 2.265 | 1.783-2.876 | 0.000 | 0.680 | 0.399-1.161 | 0.157 |
| PCI | 1.780 | 1.396-2.270 | 0.000 | 1.424 | 0.765-2.648 | 0.265 |
| COPD | 2.215 | 1.729-2.837 | 0.000 | 0.706 | 0.418-1.193 | 0.193 |
| Asthma | 2.005 | 1.464-2.745 | 0.000 | 0.917 | 0.416-2.024 | 0.831 |
| Hypertension | 1.321 | 0.867-2.014 | 0.195 | - | - | - |
| Peripheral arterial disease | 1.669 | 1.243-2.241 | 0.001 | 0.854 | 0.468-1.556 | 0.605 |
| Dyslipidemia | 2.111 | 1.676-2.659 | 0.000 | 0.648 | 0.401-1.049 | 0.078 |
| ICD | 1.753 | 0.870-3.531 | 0.116 | - | - | - |
| Pacemaker | 2.722 | 2.088-3.549 | 0.000 | 0.635 | 0.351-1.151 | 0.134 |
| Atrial fibrillation | 1.703 | 1.394-2.081 | 0.000 | 0.732 | 0.491-1.090 | 0.125 |
| Thyroid disease | 1.232 | 0.957-1.588 | 0.106 | - | - | - |
| Diabetes mellitus | 2.919 | 2.387-3.570 | 0.000 | 0.757 | 0.481-1.192 | 0.229 |
| Heart rate | 1.010 | 1.001-1.020 | 0.033 | 1.018 | 1.000-1.035 | 0.047 |
| Systolic blood pressure | 0.992 | 0.984-0.999 | 0.024 | 1.009 | 0.994-1.025 | 0.219 |
| Diastolic blood pressure | 0.945 | 0.937-0.953 | 0.000 | 0.967 | 0.946-0.989 | 0.003 |
| Fasting glucose | 1.007 | 1.004-1.009 | 0.000 | 1.003 | 0.999-1.006 | 0.104 |
| New York Heart Association class III-IV | 2.523 | 2.065-3.082 | 0.000 | 0.585 | 0.394-0.869 | 0.008 |
| eGFR | 0.975 | 0.969-0.981 | 0.000 | 0.993 | 0.981-1.004 | 0.207 |
| Leukocyte group | 1.360 | 1.241-1.491 | 0.000 |  |  | 0.073 |
| 1 |  |  |  | 0.961 | 0.534-1.732 | 0.896 |
| 2 |  |  |  | reference |  |  |
| 3 |  |  |  | 1.750 | 1.029-2.979 | 0.039 |
| 4 |  |  |  | 1.365 | 0.804-2.318 | 0.249 |
| Hemoglobin | 0.717 | 0.676-0.761 | 0.000 | 0.898 | 0.805-1.003 | 0.055 |
| BUN | 1.039 | 1.034-1.044 | 0.000 | 1.010 | 0.996-1.025 | 0.148 |
| Albumin | 0.935 | 0.902-0.970 | 0.000 | 0.984 | 0.926-1.045 | 0.599 |
| Aspirin | 0.815 | 0.665-0.998 | 0.048 | 1.216 | 0.817-1.810 | 0.334 |
| b-blocker | 1.803 | 1.355-2.398 | 0.000 | 0.525 | 0.321-0.856 | 0.010 |
| ACEi | 0.645 | 0.528-0.789 | 0.000 | 0.928 | 0.573-1.502 | 0.762 |
| ARB | 1.791 | 1.417-2.264 | 0.000 | 0.907 | 0.515-1.599 | 0.736 |
| Statin | 2.231 | 1.803-2.761 | 0.000 | 1.303 | 0.818-2.076 | 0.266 |
| Loop Diuretic | 7.081 | 5.331-9.406 | 0.000 | 0.327 | 0.192-0.556 | 0.000 |
| Thiazide Diuretics | 0.457 | 0.361-0.577 | 0.000 | 1.105 | 0.802-1.523 | 0.541 |
| Spironolactone | 1.198 | 0.981-1.464 | 0.077 | 0.940 | 0.720-1.299 | 0.653 |

**Supplemental Table 4. Subgroup analysis of Cox proportional-hazards model divided by gender for Hospitalization for heart failure. (n=2898)**

| Hospitalization for heart failure | Male |  |  | female |  |  |
| --- | --- | --- | --- | --- | --- | --- |
|  | HR | 95%CI | p-value | HR | 95%CI | p-value |
| Age | 1.009 | 0.972-1.047 | 0.636 | 1.008 | 0.970-1.048 | 0.672 |
| Race | 1.988 | 1.675-2.360 | 0.009 |  |  | 0.782 |
|  | 0.239 | 0.073-0.781 | 0.018 | 0.552 | 0.104-2.920 | 0.484 |
|  | 0.882 | 0.199-3.904 | 0.868 | 0.605 | 0.100-3.663 | 0.585 |
| BMI | 1.017 | 0.976-1.059 | 0.433 | 1.004 | 0.966-1.044 | 0.827 |
| Smoker | 1.082 | 0.439-2.670 | 0.864 | 0.978 | 0.178-5.388 | 0.980 |
| Angina pectoris | 1.628 | 0.865-3.063 | 0.131 | 2.017 | 1.046-3.891 | 0.036 |
| Stroke | 2.652 | 0.985-7.138 | 0.053 | 0.577 | 0.254-1.309 | 0.188 |
| CABG | 0.542 | 0.278-1.055 | 0.072 | 0.911 | 0.322-2.582 | 0.862 |
| PCI | 1.983 | 0.811-4.848 | 0.133 | 1.565 | 0.536-4.569 | 0.412 |
| COPD | 0.462 | 0.236-0.906 | 0.025 | 1.287 | 0.398-4.161 | 0.674 |
| Asthma | 1.566 | 0.488-5.027 | 0.451 | 0.518 | 0.153-1.753 | 0.290 |
| Peripheral arterial disease | 0.603 | 0.269-1.351 | 0.219 | 0.866 | 0.278-2.699 | 0.804 |
| Dyslipidemia | 1.034 | 0.509-2.099 | 0.927 | 0.382 | 0.186-0.783 | 0.009 |
| Pacemaker | 0.360 | 0.174-0.743 | 0.006 | 2.829 | 0.583-13.732 | 0.197 |
| Atrial fibrillation | 0.690 | 0.395-1.204 | 0.192 | 0.593 | 0.318-1.104 | 0.099 |
| Diabetes mellitus | 0.702 | 0.378-1.304 | 0.263 | 0.967 | 0.933-1.001 | 0.060 |
| Heart rate | 1.020 | 0.992-1.048 | 0.157 | 1.027 | 1.000-1.055 | 0.052 |
| Systolic blood pressure | 1.016 | 0.991-1.042 | 0.214 | 1.011 | 0.989-1.034 | 0.322 |
| Diastolic blood pressure | 0.958 | 0.925-0.992 | 0.015 | 0.967 | 0.933-1.001 | 0.060 |
| Fasting glucose | 1.005 | 1.000-1.011 | 0.035 | 0.998 | 0.992-1.005 | 0.582 |
| New York Heart Association class III-IV | 0.891 | 0.489-1.625 | 0.707 | 0.403 | 0.220-0.737 | 0.003 |
| eGFR | 0.986 | 0.970-1.001 | 0.066 | 1.002 | 0.985-1.019 | 0.838 |
| Leukocyte group |  |  | 0.054 |  |  | 0.633 |
| 1 | 0.711 | 0.293-1.726 | 0.452 | 0.909 | 0.381-2.167 | 0.830 |
| 2 | reference |  |  |  |  |  |
| 3 | 1.905 | 0.913-3.973 | 0.086 | 1.196 | 0.500-2.857 | 0.688 |
| 4 | 1.367 | 0.643-2.908 | 0.416 | 1.493 | 0.656-3.398 | 0.340 |
| Hemoglobin | 0.888 | 0.766-1.031 | 0.118 | 0.959 | 0.785-1.170 | 0.677 |
| BUN | 1.012 | 0.992-1.032 | 0.242 | 1.014 | 0.992-1.036 | 0.221 |
| Albumin | 1.030 | 0.946-1.122 | 0.493 | 0.966 | 0.872-1.071 | 0.513 |
| Aspirin | 1.511 | 0.853-2.675 | 0.157 | 1.032 | 0.547-1.946 | 0.923 |
| b-blocker | 0.571 | 0.289-1.129 | 0.107 | 0.376 | 0.163-0.870 | 0.022 |
| ACEi | 0.845 | 0.404-1.766 | 0.653 | 0.941 | 0.459-1.930 | 0.868 |
| ARB | 0.614 | 0.277-1.360 | 0.229 | 1.392 | 0.534-3.630 | 0.499 |
| Statin | 1.078 | 0.551-2.107 | 0.827 | 1.648 | 0.850-3.197 | 0.139 |
| Loop Diuretic | 0.248 | 0.106-0.580 | 0.001 | 0.448 | 0.204-0.981 | 0.045 |
| Thiazide Diuretic | 0.696 | 0.368-1.317 | 0.265 | 1.149 | 0.561-2.355 | 0.704 |
| Spironolactone | 0.733 | 0.436-1.232 | 0.241 | 0.724 | 0.401-1.307 | 0.284 |
